# Prefrontal-Hippocampal Interactions in Memory and Emotion

**DOI:** 10.3389/fnsys.2015.00170

**Published:** 2015-12-15

**Authors:** Jingji Jin, Stephen Maren

**Affiliations:** Department of Psychology and Institute for Neuroscience, Texas A&M UniversityCollege Station, TX, USA

**Keywords:** prefrontal cortex, memory, emotion, context, extinction, working memory, PTSD

## Abstract

The hippocampal formation (HPC) and medial prefrontal cortex (mPFC) have well-established roles in memory encoding and retrieval. However, the mechanisms underlying interactions between the HPC and mPFC in achieving these functions is not fully understood. Considerable research supports the idea that a direct pathway from the HPC and subiculum to the mPFC is critically involved in cognitive and emotional regulation of mnemonic processes. More recently, evidence has emerged that an indirect pathway from the HPC to the mPFC via midline thalamic nucleus reuniens (RE) may plays a role in spatial and emotional memory processing. Here we will consider how bidirectional interactions between the HPC and mPFC are involved in working memory, episodic memory and emotional memory in animals and humans. We will also consider how dysfunction in bidirectional HPC-mPFC pathways contributes to psychiatric disorders.

## Introduction

Episodic memories represent past autobiographical events and include rich details about the context in which those events occur. For example, a memory of your high school prom might include where the dance was held, when it occurred, how you traveled to the dance, and of course, who your date was for the evening. The contextual information encoded during an experience supports the later retrieval of that information, a phenomenon supported by encoding specificity (Tulving and Thomson, 1973) and *contextual retrieval* (Hirsh, 1974). That is, the content of *what* is remembered about a particular event is often critically dependent on *where* that memory is retrieved. Deficits in contextual retrieval are associated with memory impairments accompanying a variety of neural insults including age-related dementia, traumatic brain injury, stroke, and neurodegenerative disease. As such, understanding the neural circuits mediating contextual retrieval is essential for targeting interventions to alleviate memory disorders and associated cognitive impairments.

Decades of research in both humans and animals have revealed that two brain areas, the hippocampus (HPC) and medial prefrontal cortex (mPFC), are essential for the encoding and retrieval of episodic memories (Kennedy and Shapiro, 2004; Hasselmo and Eichenbaum, 2005; Diana et al., 2010; Preston and Eichenbaum, 2013). Indeed, considerable data suggests that communication between these brain areas is essential for episodic memory processes (Simons and Spiers, 2003; Preston and Eichenbaum, 2013). Anatomically, neurons in the HPC have a robust projection to the mPFC, including the infralimbic (IL) and prelimbic (PL) cortices in rats (Swanson and Kohler, 1986; Jay and Witter, 1991; Thierry et al., 2000; Varela et al., 2014). In primates, there are projections that originate from hippocampal CA1 and terminate in the orbital and medial frontal cortices (areas 11, 13, 14c, 25 and 32; Zhong et al., 2006). These PFC connectivity patterns seem to be similar in humans and monkeys; for example, both humans and monkeys have fimbria/fornix fibers (which originate from the hippocampus and subiculum) terminating in the medial orbital PFC (Cavada et al., 2000; Croxson et al., 2005). For these reasons, models of episodic retrieval have largely focused on the influence of contextual representations encoded in the HPC on memory retrieval processes guided by the mPFC (Maren and Holt, 2000; Hasselmo and Eichenbaum, 2005; Ranganath, 2010). Yet emerging evidence suggests that the mPFC itself may be critical for directing the retrieval of context-appropriate episodic memories in the HPC (Navawongse and Eichenbaum, 2013; Preston and Eichenbaum, 2013). This suggests that indirect projections from the mPFC to HPC may be involved in episodic memory, including contextual retrieval (Davoodi et al., 2009, 2011; Hembrook et al., 2012; Xu and Südhof, 2013). Moreover, abnormal interactions between the HPC and mPFC are associated with decreased mnemonic ability as well as disrupted emotional control, which are major symptoms of psychiatric disorders such as schizophrenia, depression, specific phobia, and post-traumatic stress disorder (PTSD; Sigurdsson et al., 2010; Godsil et al., 2013; Maren et al., 2013). Here we will review the anatomy and physiology of the HPC-mPFC pathway in relation to memory and emotion in an effort to understand how dysfunction in this network contributes to psychiatric diseases.

## Anatomy and Physiology of Hippocampal-Prefrontal Projections

It has long been appreciated that there are both direct monosynaptic projections, as well as indirect polysynaptic projections between the HPC and the mPFC (Hoover and Vertes, 2007). In rats, injections of retrograde tracers into different areas of the mPFC robustly label neurons in the VH and subiculum (Jay et al., 1989; Hoover and Vertes, 2007). In addition, injections of the anterograde tracer, *Phaseolus vulgaris*-leucoagglutinin (PHA-L), into the HPC reveal direct projections to the mPFC (Jay and Witter, 1991). Hippocampal projections to the mPFC originate primarily in ventral CA1 and ventral subiculum; there are no projections to the mPFC from the dorsal hippocampus or dentate gyrus. Therefore, the direct functional interactions we discuss below focus on ventral hippocampal and subicular projections to the mPFC. Hippocampal projections course dorsally and rostrally through the fimbria/fornix, and then continue in a rostro-ventral direction through the septum and the nucleus accumbens (NAcc), to reach the IL, PL, medial orbital cortex, and anterior cingulate cortex (Jay and Witter, 1991; Cenquizca and Swanson, 2007). Afferents from CA1 and the subiculum are observed throughout the entire rostro-caudal extent of the mPFC, with only sparse projections to the medial orbital cortex.

**Figure 1:**
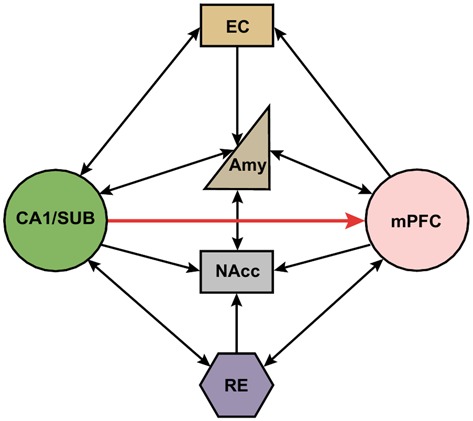
**Schematic representation of direct and indirect neural circuits between the medial prefrontal cortex and hippocampus/subiculum.** Hippocampal area CA1 and the subiculum (SUB) have strong direct projections to the mPFC, but there are no direct projections from the mPFC back to the HPC. The reuniens (RE) and amygdala has reciprocal connections with both the mPFC and HPC. NAcc receives inputs from mPFC, HPC, RE and amygdala. mPFC also project to entorhinal cortex (EC) which in turn has reciprocal projections with HPC. SUB, subiculum; EC, entorhinal cortex; Amy, amygdala; NAcc, nucleus accumbens; RE, nucleus reuniens; mPFC, medial prefrontal cortex.

Indirect multi-synaptic pathways from the HPC to mPFC include projections through the NAcc and ventral tegmental area (VTA), amygdala, entorhinal cortex (EC), and midline thalamus (Maren, 2011; Russo and Nestler, 2013; Wolff et al., 2014). These complex multi-synaptic pathways from both subcortical and cortical areas are critically involved in higher cognitive functions that are related to several major psychiatric disorders. For example, it has been reported that NAcc receives convergent synaptic inputs from the PFC, HPC and amygdala (Groenewegen et al., 1999). This cortical-limbic network has been shown to mediate goal-directed behavior by integrating HPC-dependent contextual information and amygdala-dependent emotional information with cognitive information processed in the PFC (Goto and Grace, 2005, 2008). In addition, the mPFC projects to the thalamic nucleus reuniens (RE), which in turn has dense projections to the HPC (Varela et al., 2014). Importantly, this projection is bidirectional, which provides another route for the HPC to influence the mPFC (Figure 1). Interestingly, it has been shown that single RE neurons send collaterals to both the HPC and mPFC (Hoover and Vertes, 2012; Varela et al., 2014). This places the RE in a key position to relay information between the mPFC and HPC to coordinate their functions (Davoodi et al., 2009, 2011; Hembrook et al., 2012; Hoover and Vertes, 2012; Xu and Südhof, 2013; Varela et al., 2014; Griffin, 2015; Ito et al., 2015). The mPFC also has strong projections to the EC, which in turn has extensive reciprocal connections with hippocampal area CA1 and the subiculum (Vertes, 2004; Cenquizca and Swanson, 2007). Interestingly, the CA1 and subiculum send direct projections back to the mPFC, allowing these areas to form a functional loop that enables interactions between cortical and subcortical areas during memory encoding and retrieval (Preston and Eichenbaum, 2013).

The physiology of projections between the HPC and mPFC has been extensively investigated in rodents. These projections consist of excitatory glutamatergic pyramidal neurons that terminate on either principle neurons or GABAergic interneurons within the mPFC (Jay et al., 1992; Carr and Sesack, 1996; Tierney et al., 2004). Electrical stimulation in hippocampal area CA1 or the subiculum produces a monosynaptic excitatory postsynaptic potential (EPSP) followed by fast and slow inhibitory postsynaptic potentials (IPSPs); the latter are due to both feedforward (Jay et al., 1992; Tierney et al., 2004) and feedback inhibition (Dégenètais et al., 2003). Excitatory responses evoked in mPFC neurons by electrical stimulation of the HPC are antagonized by CNQX but not by AP5, indicating that these responses are AMPA-receptor dependent (Jay et al., 1992). Hippocampal synapses in the mPFC exhibit activity-dependent plasticity including long-term potentiation (LTP), long-term depression (LTD), and depotentiation (Laroche et al., 1990, 2000; Jay et al., 1996; Burette et al., 1997; Takita et al., 1999). These forms of plasticity are NMDA receptor-dependent and involve activation of serine/threonine kinases such as CaMKII, PKC, and PKA (Dudek and Bear, 1992; Bliss and Collingridge, 1993; Jay et al., 1995, 1998; Burette et al., 1997; Takita et al., 1999).

Within the indirect mPFC-RE-HPC pathway, a large proportion of RE projection neurons are glutamatergic (Bokor et al., 2002). RE stimulation produces strong excitatory effects on both HPC and PFC neurons (Dolleman-Van der Weel et al., 1997; Bertram and Zhang, 1999; McKenna and Vertes, 2004), suggesting that the RE is capable of modulating synaptic plasticity in both the HPC and mPFC (Di Prisco and Vertes, 2006; Eleore et al., 2011).

## Working Memory

Both the HPC and mPFC have been implicated in working memory and mounting evidence suggests that communication between these two structures is critical for this process. Working memory is a short-term repository for task-relevant information that is critical for the successful completion of complex tasks (Baddeley, 2003). For example, in a spatial working memory task, animals must hold in memory the location of food rewards to navigate to those locations after a delay. Disconnection of the HPC and mPFC with asymmetric lesions disrupts spatial working memory (Floresco et al., 1997; Churchwell and Kesner, 2011). PFC lesions disrupt the spatial firing of hippocampal place cells whereas HPC lesions disrupt anticipatory activity of mPFC neurons in working memory tasks (Kyd and Bilkey, 2003; Burton et al., 2009). This suggests that interactions between the HPC and mPFC are crucial for this form of memory.

One index of the functional interaction of different brain regions is the emergence of correlated neural activity between them during behavioral tasks. For example, simultaneous recordings in the HPC and mPFC reveal synchronized activity during working memory tasks (Jones and Wilson, 2005; Siapas et al., 2005; Benchenane et al., 2010; Hyman et al., 2010). Hippocampal theta oscillations (4~10 Hz), which are believed to be important in learning and memory, are phase-locked with both theta activity and single-unit firing in the mPFC (Siapas et al., 2005; Colgin, 2011; Gordon, 2011). Medial prefrontal cortex firing lags behind the hippocampal LFP, suggesting that information flow is from the HPC to mPFC (Hyman et al., 2005, 2010; Jones and Wilson, 2005; Siapas et al., 2005; Benchenane et al., 2010; Sigurdsson et al., 2010). Interestingly, this synchronized activity is not static, but is modulated during tasks associated with working memory or decision-making. Recently, Spellman et al. (2015) used optogenetic techniques to manipulate activity in the VH-mPFC pathway during a spatial working memory task (Spellman et al., 2015). They found that in a “four-goal T-maze” paradigm, direct projections from the VH to the mPFC are crucial for encoding task-relevant spatial cues, at both neuronal and behavioral levels. Moreover, gamma activity (30~70 Hz) in this pathway is correlated with successful cue encoding and correct test trials and is disrupted by VH terminal inhibition. These findings suggest a critical role of the VH-mPFC pathway in the continuous updating of task-related spatial information during spatial working memory task.

Indirect projections from the mPFC back to the HPC are also involved in working memory. For example, lesions or inactivation of the RE cause deficits in both radial arm maze performance and a delayed-non-match-to-position task that has previously been shown to be dependent on both the HPC and mPFC. This suggests that the RE is required for coordinating mPFC-HPC interactions in working memory tasks (Porter et al., 2000; Hembrook and Mair, 2011; Hembrook et al., 2012). Recently, Ito et al. (2015) proposed that the mPFC→RE→sHPC projection is also crucial for representation of the future path during goal-directed behavior. Therefore, the RE is considered to be a key relay structure for long-range communication between cortical regions involved in navigation (Ito et al., 2015). Hence, both direct and indirect connections between the HPC and mPFC contribute to the hippocampal-prefrontal interactions important for working memory processes as well as spatial navigation.

## Episodic Memory

Episodic memory is a long-term store for temporally dated episodes and the temporal-spatial relationships among these events (Tulving, 1983). These memories contain “what, where and when” information that place them in a spatial and temporal context. Although animals cannot explicitly report their experience, their knowledge of “what, where and when” information suggests that they also use episodic memories (Eacott and Easton, 2010). For example, animals can effectively navigate in mazes that require them to remember “what-where” information that is coupled to time (“when”; Fouquet et al., 2010). Considerable work indicates that the HPC and mPFC are critically involved in encoding and retrieval of episodic-like memories (Wall and Messier, 2001; Preston and Eichenbaum, 2013). Within the hippocampal formation (HPC), the perirhinal cortex (PRh) is thought to be crucial in signaling familiarity-based “what” information, whereas the parahippocampal cortex (PH) is involved in processing “where” events occur (Eichenbaum et al., 2007; Ranganath, 2010). Both the PRh and PH are connected with the EC, which in turn has strong reciprocal projections with the HPC and subiculum; this provides an anatomical substrate for the convergence of “what” and “where” information in the HPC (Ranganath, 2010). In support of this idea, studies have shown that hippocampal networks integrate non-spatial and spatial/contextual information (Davachi, 2006; Komorowski et al., 2009). More recently, there is evidence that neurons in hippocampal CA1 code both space and time, allowing animals to form conjoint spatial and temporal representations of their experiences (Eichenbaum, 2014). These findings suggest a fundamental role of the HPC for encoding episodic memories.

There is also considerable evidence that the mPFC contributes to episodic memory through cognitive or strategic control over other brain areas during memory retrieval. Although prefrontal damage does not yield severe impairments in familiarity-based recognition tests (Swick and Knight, 1999; Farovik et al., 2008), impairments are observed in tasks that require recollection-based memory, which rely on the retrieval of contextual and temporal information and resolution of interference (Shimamura et al., 1990, 1995; Dellarocchetta and Milner, 1993; Simons et al., 2002). It has been suggested that the mPFC is important for the integration of old and new memories that share overlapping features, whereas the HPC is more important in forming new memories (Dolan and Fletcher, 1997). These findings suggest that there is functional dissociation between the mPFC and HPC during episodic memory encoding and retrieval in some cases. However, these two structures interact with each other in order to complete memory tasks that require higher levels of cognitive control.

In line with this idea, human EEG studies have shown that HPC-mPFC synchrony is associated with memory recall. For instance, encoding of successfully recalled words was associated with enhanced theta synchronization between frontal and posterior regions (including parietal and temporal cortex), indicating that the interaction between these two areas is involved in memory encoding (Weiss and Rappelsberger, 2000; Weiss et al., 2000; Summerfield and Mangels, 2005). Furthermore, depth recordings in epilepsy patients reveal theta-oscillation coherence between the medial temporal lobe and PFC during verbal recall tests, suggesting that synchronized neural activity is involved in the encoding and retrieval of verbal memory (Anderson et al., 2010). Recently, work in monkeys has revealed that different frequency bands within the HPC and mPFC have different functional roles in object-paired associative learning (Brincat and Miller, 2015). Collectively, these data indicated that the HPC and mPFC interactions are dynamic during episodic memory encoding and retrieval.

## Contextual Memory Retrieval

When humans and animals form new memories, contextual information associated with the experience is also routinely encoded without awareness (Tulving and Thomson, 1973). Contextual information plays an important role in memory retrieval since the content of *what* is often critically dependent on *where* that memory is retrieved (Hirsh, 1974; Maren and Holt, 2000; Bouton, 2002). This “contextual retrieval” process allows the meaning of a cue to be understood according to the context in which it is retrieved (Maren et al., 2013). For example, encountering a lion in the wild might be a life-threatening experience to someone, but seeing the same lion kept in its cage in the zoo might be an interesting (and non-threatening) experience. Therefore, the same cue in different contexts has totally different meanings. Contextual processing is highly adaptive because it resolves ambiguity during memory retrieval (Bouton, 2002; Maren et al., 2013; Garfinkel et al., 2014). Decades of research in both humans and animals have revealed that the HPC and mPFC are essential for contextual retrieval (Kennedy and Shapiro, 2004; Hasselmo and Eichenbaum, 2005; Diana et al., 2010; Maren et al., 2013). Humans and animals with disconnections in the HPC-mPFC network have deficits in retrieving memories that require either source memories or contextual information (Schacter et al., 1984; Shimamura et al., 1990; Simons et al., 2002). Thus, these brain regions are key components of a brain circuit involved in episodic memory, and connections between them are thought to support contextual retrieval.

Contextual retrieval is also critical for organizing defensive behaviors related to emotional memories (Bouton, 2002; Maren and Quirk, 2004; Maren et al., 2013). Learning to detect potential threats and organize appropriate defensive behavior while inhibiting fear when threats are absent are highly adaptive functions linked to emotional regulation (Maren, 2011). Deficits in emotional regulation often result in pathological fear memories that can further develop into fear and anxiety disorders, such as PTSD (Rasmusson and Charney, 1997). Studies indicate that fear memories are rapidly acquired and broadly generalized across contexts. In contrast, extinction memories often yield transient fear reduction and are bound to the context in which extinction occurs (Bouton and Bolles, 1979; Bouton and Nelson, 1994). After extinction, fear often relapses when the feared stimulus is encountered outside the extinction context-a phenomenon called fear “renewal” (Bouton, 2004; Vervliet et al., 2013).

Recent work indicates that the HPC-mPFC network plays a critical role in regulating context-dependent fear memory retrieval after extinction (Maren and Quirk, 2004; Maren, 2011; Orsini and Maren, 2012; Maren et al., 2013; Jin and Maren, 2015). Disconnection of the VH from the mPFC impairs fear renewal after extinction (Hobin et al., 2006; Orsini et al., 2011). Inactivation of the VH also modulates the activity of both interneurons and pyramidal neurons in the PL, and influences the expression of fear behavior in extinguished rats (Sotres-Bayon et al., 2012). Moreover, VH neurons projecting to both the mPFC and amygdala are preferentially involved in fear renewal (Jin and Maren, 2015), suggesting that VH might modulate memory retrieval by coupling activity in the mPFC and amygdala. Ultimately, the hippocampus appears to gate reciprocal mPFC-amygdala circuits involved in the expression and inhibition of fear (Herry et al., 2008; Knapska and Maren, 2009; Knapska et al., 2012). It has also been shown that the vmPFC-HPC network is involved in the context-dependent recall of extinction memories in humans (Kalisch et al., 2006; Milad et al., 2007). These observations support the idea that the HPC-mPFC pathway is critically involved in the context-specificity of fear memories, whereby the transmission of contextual information from the HPC to the mPFC generates context-appropriate behavioral response by interacting with the amygdala.

In animals, extinction learning induces a potentiation of VH-evoked potentials in the mPFC, while low frequency stimulation of the VH disrupts this potentiation and prevents extinction recall (Garcia et al., 2008). Chronic stress impairs the encoding of extinction by blocking synaptic plasticity in the HPC-mPFC pathway (Garcia et al., 2008; Maren and Holmes, 2015). In addition, it has been shown that brain-derived neurotrophic factor (BDNF) in the VH-IL pathway is involved in extinction learning (Peters et al., 2010; Rosas-Vidal et al., 2014). Finally, histone acetylation in the HPC-IL network influences extinction learning (Stafford et al., 2012). These findings indicate that interactions between the HPC and mPFC are critical for encoding extinction memories.

## HPC-PFC Interaction and Psychiatric Disorders

Abnormal functional interactions between the HPC and mPFC have been reported in several psychiatric disorders. For example, patients with schizophrenia exhibit aberrant functional coupling between the HPC and mPFC during rest and during working memory performance (Meyer-Lindenberg et al., 2005; Zhou et al., 2008; Lett et al., 2014). This has been confirmed in animal models of schizophrenia, which also exhibit impaired working memory as well as decreased hippocampal-prefrontal synchrony (Sigurdsson et al., 2010). Abnormal interaction between the HPC and mPFC also causes deficits in emotional regulation associated with psychiatric disorders. Considerable evidence associates major depressive disorders with structural changes as well as functional abnormalities in hippocampal-prefrontal connectivity in both animals and humans (Bearden et al., 2009; Genzel et al., 2015). For example, the HPC and mPFC exhibit increased synchrony in anxiogenic environments (Adhikari et al., 2010; Schoenfeld et al., 2014; Abdallah et al., 2015). Moreover, traumatic experiences and pathological memories are linked to abnormal hippocampal-prefrontal interactions in PTSD patients, which in turn are associated with impaired contextual processing that mediates emotional regulation (Liberzon and Sripada, 2007). These seemingly distinct psychiatric disorders share similar symptoms: dysregulated interactions between the HPC and mPFC may be common to this shared symptomatology. Thus, the neural network between the HPC and mPFC is a promising target for future therapeutic interventions associated with these psychiatric disorders.

## Conclusion

Animal and human studies strongly implicate the HPC-mPFC network in cognitive process and emotional regulation associated with psychiatric disorders such as schizophrenia, anxiety disorders, and PTSD. Physical or functional disruptions in the HPC-mPFC circuit might be a form of pathophysiology that is common to many psychiatric disorders. Further, study of the physiology and pathophysiology of hippocampal-prefrontal circuits will be essential for developing novel therapeutic interventions for these diseases.

## Funding

Supported by a grant from the National Institutes of Health (R01MH065961) and a McKnight Memory and Cognitive Disorders Award to SM.

## Conflict of Interest Statement

The authors declare that the research was conducted in the absence of any commercial or financial relationships that could be construed as a potential conflict of interest.

## Abbreviations

EC: entorhinal cortex
HPC: hippocampus
IL: infralimbic prefrontal cortex
mPFC: medial prefrontal cortex
NAcc: nucleus accumbens
PH: parahippocampal cortex
PL: prelimbic prefrontal cortex
PRh: perirhinal cortex
PTSD: post-traumatic stress disorder
RE: nucleus reuniens
SUB: subiculum
VH: ventral hippocampus
vmPFC: ventromedial prefrontal cortex
VTA: ventral tegmental area.
